# A Pre-Embolic State

**Published:** 1879-02

**Authors:** Ephraim Cutter

**Affiliations:** 20 Tremont Temple, Boston, Mass.


### ARTICLE V.

### A PRE-EMBOLIC STATE. By EPHRAIM CUTTER, M.D., Boston, Mass.

Instances of puerperal embolism resulting in unexpected and instant death are not uncommon. This embolism is a condition in which a vascular clot plugs up a blood vessel, the plug being called an embolus. Thrombi are clots that have not become plugs or emboli. To Dr. Salisbury we are indebted for our investigation of the causes of thrombi.

According to the latter, filaments of fibrin forming clots differ from ordinary fibrin filaments. He has pointed these out in consumptive blood, and I have photographed and demonstrated the same in cases in which I have sought them. The latter may be of the same chemical nature but their morphology and genesis differ. The consumptive fibrin filaments of Salisbury are minute, straight and transparent, crossing each other at acute angles, radiating from centers and interlacing like a spider's web. They exist in health but in such surpassing minuteness and transparency as to elude all but the very best objectives and observations. Whereas the consumptive fibrin filaments are large, coarse and easily detected.

If any one will anæsthetize a frog, open the abdominal cavity, expose the spleen, clip away with scissors a minute section and place it under an objective having a magnifying power of 400 diameters, he will see these fibrin cells spinning the beautiful filaments described by our author ten years ago, which observations I have myself verified and am prepared to verify. But these are quite different from those found in thrombi or, if preferred "emboli." They are loose, white, opaque, rough, flat, of sensible diameter, and arranged, not in rectilinear lines but in skeins curled up like a housewife's mat or a sailor's fancy coil of rope. They appear like filaments of albumen coagulated by some mineral acid. They are readily found in old rheumatic cases, especially when cardiac complication is present. They have also been discovered by Dr. Salisbury and demonstrated by him to me, in the blood of persons living exclusively or immoderately upon certain articles of food, as strawberries.

Now, calling the state in which clots are found in the heart or blood vessels, embolism, the concretions being so large as to be visible to the naked eye and to block up mechanically the caliber of the blood channels, if these emboli can be detected before the occurrence of embolism, we shall be able to detect a pre-embolic state with thrombi so minute as to be recognizable only by microscopic examination of the blood.

This is what Dr. Salisbury has done; and he states that he has found these clots in the morphological elements of the blood a long time previous to the plugging of blood vessels.

The treatment he directs is restorative to what he calls the "parent glands," and causes them to organize their products more perfectly. This is largely effected by regulating the alimentation. The micrography of the blood tells how the general condition improves.

*Remarks:* Notice that it is not here intended to teach that in all cases this pre-embolic state occurs, but that in all cases examined in the pre-embolic stage he has found the minute thrombi. I also have found them. It is quite another thing to say that they will always be found by other observers. I am not aware of any mention of this fact or of its use in medical practice save by the discoverer and his pupils.

Properly speaking an embolus is a clot that acts as a plug. Thrombi are clots that have not become emboli. I took my cue from Dr. Sullivan, and think no harm is done as long as the thing intended is understood. I close with a brief quotation from the *Microscopic Examination of Blood*, by *J. M. Salisbury*, M. D., New York, Moorhead, Bond & Co., 1868, page 22:

" Whenever there exists in the blood, abnormal bodies that are insoluble in the serum, more or less irritation of the lining tissue of the blood apparatus, especially in the heart and vicinity, is the result. Sooner or later, the organic muscular fibers lose a part of their tonicity, and there are frequently tired feelings, and wandering pains and aches, which are more likely to hang about the cardiac region than elsewhere. The insoluble matters floating in the blood have a tendency to fix themselves in or near the heart, to the epithelial lining, the secretions from which assume a more or less plastic and sticky condition from the irritation excited. These fixed particles become centers for the gradual accretion of fibrin, which applies itself slowly, layer upon layer, and finally little by little we have formed thrombi. These from time to time break loose from their points of attachment and float in the blood stream as emboli. There is another condition of the blood elements where there is great danger of thrombosis and embolism, and which may be readily diagnosticated by means of the microscope, in time to avert the serious results which may await the patient. *This is stickiness and plasticity of the fibrin filaments* and colorless corpuscles. This stickiness often extends to the blood discs. Whenever this state is present the colorless globules are found—instead of being scattered about singly—

sticking to each other in groups of two, three, four or more. The fibrin filaments are also sticky and plastic, and impede the free flow of the blood cells through their net-work. The result is a marked tendency to the formation of thrombi and emboli. Emboli under such conditions are produced frequently, simply by the sticking together of the colorless corpuscles, forming masses of greater or less size. They are also formed by the breaking loose of thrombi. This sticky state of the blood is usually scorbutic, arising mainly from deranged or defective alimentation."

20 TRÉMONT TEMPLE, Boston, Dec. 21st, 1878.

---

FISH-BONES IN THE PHARYNX.—When a fish-bone gets lodged in the throat, Prof. Voltolini thinks the worst thing a physician unpracticed with the laryngoscope can do is, to try to push the foreign body down into the stomach. This manœuvre never succeeds, but simply wedges the bone tightly in between the folds of the mucous membrane. Voltolini, therefore, suggests under such circumstances to let the fish-bone alone until a physician be found able to extract it, under guidance of the throat mirror. But, in the meantime, he advises the use of diluted hydrochloric acid as a gargle. He found, in a solution of 4.0 hydrochloric, or nitric acid, 1 to 240.0 water, the hardest fish-bones become soft and flexible in one hour, thinner bones were like threads after one half hour. And he thinks the continued gargling with the above mixture (the teeth being protected by oil or lard) will have the same effect upon a fish-bone lodged in the pharynx.—(Monatsschrift f. Ohrenheilk.)

THE office of the Illinois State Board of Health has been removed to the State House in Springfield. All communications should be addressed to Dr. A. L. Clark, Secretary of the Board, Springfield, Ill.

